# Incidental diagnosis of a large cardiac thrombus swinging through an interatrial communication in a COVID-19 patient: Case report and literature review

**DOI:** 10.1016/j.amsu.2021.102967

**Published:** 2021-10-19

**Authors:** Samia Berrichi, Zakaria Bouayed, Karima Benbouchta, Amine Kossir, Houssam Bkiyar, Nabila Ismaili, Noha El Ouafi, Brahim Housni

**Affiliations:** a Anesthesia and Resuscitation Department, MOHAMMED VI University Hospital Center, Oujda, Morocco; b Cardiology Department, Mohammed VI University Hospital, Oujda, Morocco; c Simulation Center, Faculty of Medicine and Pharmacy, Oujda, Morocco; d Laboratory of Epidemiology, Clinical Research, and Public Health, Faculty of Medicine and Pharmacy, Oujda, Morocco

**Keywords:** COVID-19, Cardiac thrombosis, Interatrial shunt

## Abstract

**Introduction:**

The hypercoagulability state induced by COVID-19 has been well established and various forms of subsequent thromboembolic events have been reported throughout literature including multiple cases of intracardiac thrombi, four of which in our center alone, this case being the fifth.

**Case report:**

We report the case of a 38-year-old male with no prior cardiovascular history who -subsequently to a COVID-19 infection-developped a right atrial thrombosis associated to a pulmonary embolism, and in whom cardiography revealed an interatrial communication. Management relied upon curative doses of low molecular weight heparin (LMWH) with favourable outcome.

**Discussion:**

In our discussion, we lay out the various physiopathological mechanisms incriminated throughout literature in the genesis of a hypercoagulability state distinctive of COVID-19, before highlighting the incidence of an interatrial communication (whether a Potent Foramen Ovale or Atrial Septal Defect) discovered in patients with COVID-19, and the potential paradoxical embolization risks they imply as well as reported cases. A mention of hemostatic parameters monitored was also warranted. Finally we discuss the guidelines in terms of prophylactic and therapeutic anticoagulation in hospitalized patients before discussing cardiac thrombosis's therapeutic options.

**Conclusion:**

Our case highlights various key points which could change the prognosis of COVID-19 patients, whether related to the underdiagnosis of interatrial abnormalities or with regards to the diagnosis to thromboembolic events, but also the indisputable place of anticoagulation in COVID-19 management.

## Highlights

• Thromboembolic events complicating COVID-19 have been well described and are attributed to a hypercoagulability state distinctive of COVID-19.
• A pre-existing Potent Foramen Ovale (PFO) or Atrial septal Defect (ASD) in COVID-19 patients is unknown as only a handful of studies covered the subject.
• The association of a cardiac thrombosis with a pulmonary embolism and interatrial communication exposes patients to risks of paradoxical embolization and subsequent arterial thrombotic events.
• Prophylactic and curatrive anticoagulation is unrefutably a key pillar in the management of COVID-19.

## 1 Introduction

After a year and half since the first cluster of COVID-19 cases have been reported in Wuhan, and now with more than 177 million cases worldwide confirmed to this day [1] and hundreds of cases published, it has become clear that COVID-19 is a complex multisystemic disease involving many organs and body systems [2].

The thromboembolism events induced by COVID-19 and their consequences in terms of morbidiy and mortality have been well documented [3], and are even greater when occurring in patients with an interatrial communication on account of the paradoxical embolization risks they entail.

Prophylactic anticoagulation has been recommended in all hospitalized patients admitted for management of COVID-19 specifically to lower the chances of thromboenbolic events and reduce their mortality [4].

We report the case of a 38-year-old male with a COVID-19 infection and recent rapid worsening of an acute respiratory distress diagnosed with a pulmionay embolism and in whom cardiography revealed an interatrial communication, managed with curative doses of low molecular weight heparin (LMWH) with favourable outcome.

This paper has been reported in line with the SCARE 2020 criteria [5].

## 2 Case report

A 38-year-old man with a history of schizophrenia under Haloperidol, Trihexyphenidyl, and Levomepromazine was admitted to the ER for the management of an acute respiratory distress, history of the present illness revealed that the patient first developed 2 weeks prior to his admission a flu-like syndrome made of fever, shivering, dry cough, myalgia, and shortness of breath on exertion for which the patient self-medicated without any improvement.

Following the rapid worsening of his dyspnea a day before, the patient sought the ER. On initial clinical assessment he was conscious and well oriented in time and space with a Glasgow Scale of 15/15, afebrile (36.8 °C), tachycardic at a 140 beat per minute, normotensive at 125/75 mmHg, with an O_2_ saturation of 84% on ambient air and 91% under nasal oxygen therapy (3L/min), pulmonary auscultation revealed right basal crackles.

**Fig. 1:**
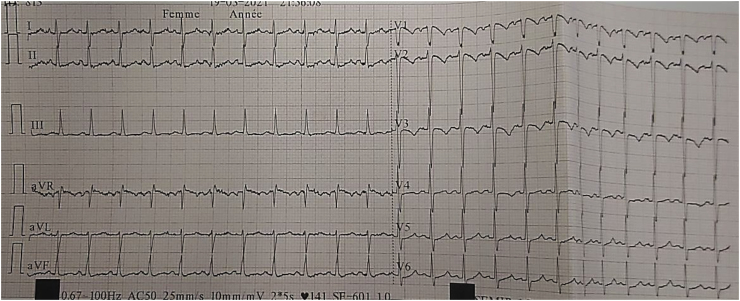
EKG showing a sinus rhythm with HB of 140bpm, an incomplete RBBB, and T wave inversion in V1 to V3.

An electrocardiogram (EKG) revealed a sinus rhythm with a high frequency of 140 beat per minute with an incomplete Right Bundle Branch Block (RBBB) and T wave inversion in V1 to V3 (Fig. 1).

Laboratory tests revealed a slightly elevated WBC (White Blood Count) of 11150/μL, a CRP (C Reactive Protein) level of 193,29 mg/L, with a hemoglobin at 11,1 g/dl, serum ferritin level of 1017,64 ng/mL, and LDH levels of 641 IU/L, HS Troponin levels at 115,2 ng/L, and an IL-6 level of 61 pg/mL, a platelet count of 239,000, D-dimers levels at 3293 ng/mL, normal electrolyte, liver and kidney function tests. A SARS-Cov-2 RT-PCR of a nasal swap sample came back positive.

**Fig. 2:**
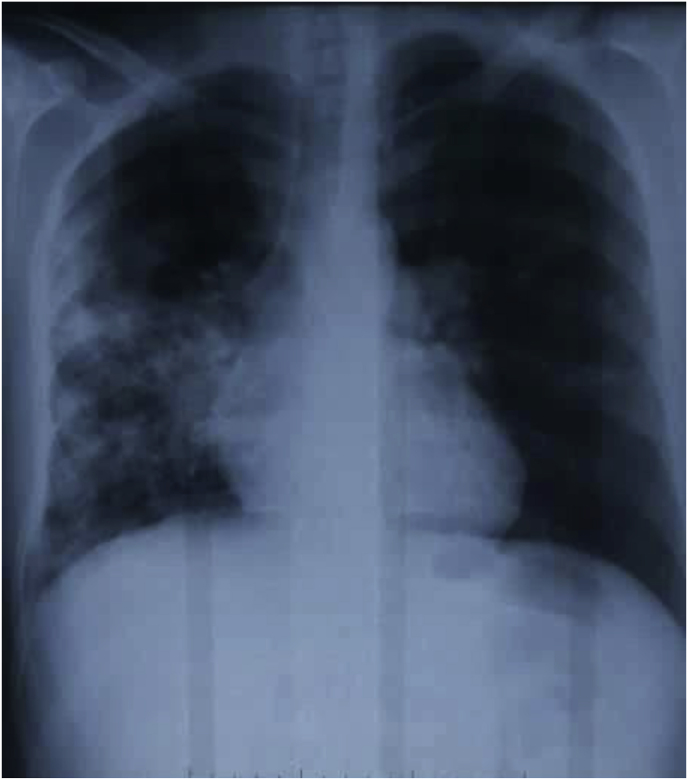
CXR showing ground glass opacities in the mid and lower zones of the right lung.

**Fig. 3:**
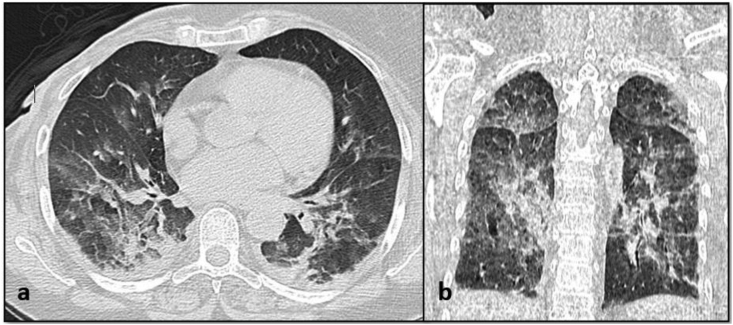
Axial (a) and Coronal (b) lung windows showing bilateral multifocal subpleural and peribronchial GGO with septal thickening and consolidation mainly in the periphery.

**Fig. 4:**
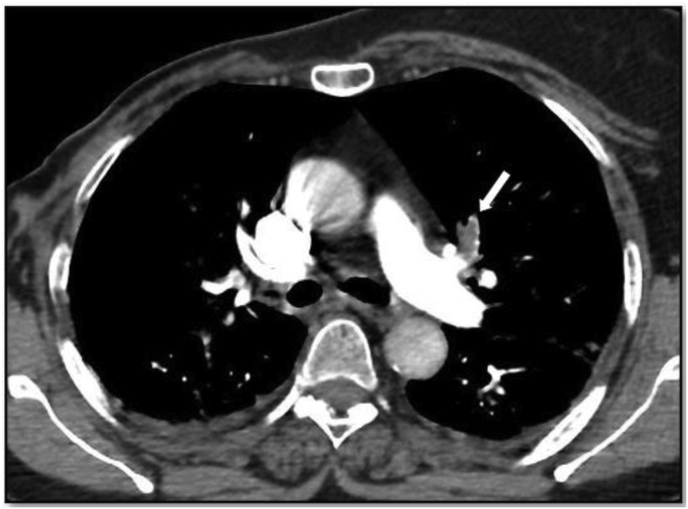
Axial sequence of a contrast-enhanced CT in mediastinal window showing a filling defect (arrow) in the left lobar pulmonary artery suggestive of pulmonary embolism.

Initial arterial blood gas revealed: 7,54 pH, 116 mmHg PaO2, 23 mmHg PaCO2, 20mEq/l HCO3-, 2,29mmol/l lactatemia. A CXR showed ground-glass opacities (GGO) in the right lung (Fig. 2), Chest CT showed bilateral multifocal subpleural and peribronchial GGO (Glass Ground Opacities) with septal thickening and consolidation mainly in the periphery (Fig. 3), contrast-enhanced sequences revealed a filling defect in the left lobar pulmonary artery suggestive for pulmonary embolism (Fig. 4).

**Fig. 5:**
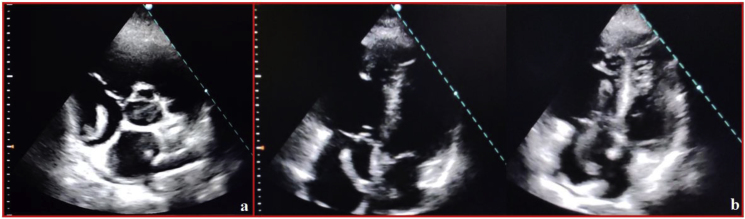
TTE para-sternal short axis view (a) and apical four-chamber view (b) showing a large thrombus inside the right cavities protruding into the left atrium.

**Fig. 6:**
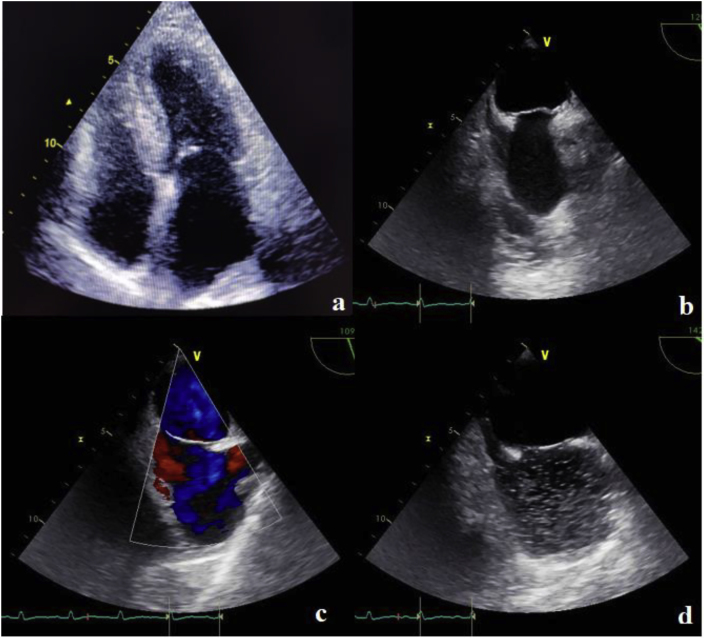
Six weeks Follow-up TTE apical four-chamber view (a) showing a normalization of the right heart cavities completed by a TEE (b) showing no septal aneurysm, ASD, nor a PFO. No right-to-left shunt on doppler flow (c) as well as a negative bubble test (d).

Considering the rapid worsening of the dyspnea, the EKG abnormalities and the pulmonary embolism we performed a transthoracic echocardiography (TTE) unveiling a large serpiginous floating thrombus (90 × 15mm) in the right and left atrium, straddling across an interatrial communication, and extending across the tricuspid and mitral valves, into the respective ventricles, the right heart cavities were dilated with paradoxical motion of the ventricular septum as well as a severe right ventricular systolic dysfunction with classic McConnell's sign and moderate pulmonary hypertension (pulmonary artery systolic pressure was estimated as 50 mmHg) (Figs. 5 and 6). Transesophageal echocardiography (TEE) was not performed given the patient's respiratory distress.

For the COVID-19 pneumonia the patient was treated with ceftriaxone 2g/day and levofloxacin 500mg/12h during ten days, dexamethasone 6mg/d, vitamins C 2g/12h and D 25000IU/week, zinc 45mg/day, and aspirin 160mg/day for the entire duration of hospitalization. As for the intra-cardiac thrombus and pulmonary embolism he received an 8000 IU subcutaneous injection of sodic enoxaparin every 12h also for the duration of his hospitalization.

A follow-up TTE was performed after 6 days revealing the total disappearance of the thrombus. Even though the patient exhibited no acute symptoms suggestive of a thromboembolic event a whole-body contrast-enhanced CT was performed as a precautionary measure and came back normal except for the pre-existing pulmonary embolism.

Follow-up laboratory tests showed a decrease of the inflammatory markers as well as D-dimers (from 3293 to 748 ng/mL).

Ther patient was gradually weaned off oxygen and discharged 14 days after admission, upon discharge we prescribed Apixaban 5mg/12h in concertation with our center's cardiology department which scheduled follow-up appointment for treatment adjustment and management of the interatrial communication.

The patient was seen 6 weeks later, a TTE was performed revealing a complete resolution of the pulmonary hypertension with normalization of the right heart cavities, completed by a TEE which showed no septal aneurysm, ASD, nor a PFO completed by a bubble test that was negative (Fig. 6).

In retrospect, we retained the diagnosis of a PFO re-opened by the right-heart high pressure secondary to the pulmonary embolism which progressed towards a functional closure of the PFO after normalization of the right atrial pressure.

## 3 Discussion

Multiple cases of thromboembolic events related to COVID-19 have been reported, varying in terms of localisation (pulmonary, cardiac, peripheral arterial and/or venous), extension, gravity, and clinical features.

The high frequency of reported COVID-19 related thromboembolic events [6,7] raises the question of a unique physiopathology [8,9]. Various intrigued pathological phenomena were incriminated [10]: from the cytokin storm [11], passing by the complement cascade activation [12], the macrophage activation [13] and antiphospholipid antibody [14] syndromes, to the Renin angiotensin system overactivation [15]. All contributing to the genesis of a hypercoaguability state.

We report the case of a 38-year-old male with no cardiovacular risk factors other than his gender and no cardiovascular history who developped a COVID-19 pneumonia complicated shortly after with a large right intraatrial thrombus associated to a left lobar pulmonary artery, subsequently the patient's dyspnea worsened rapidly. The contrast-enhanced CT revealed a pulmonary embolism while the TTE not only unveiled a large thrombus inside the right atrium but also an interatrial communication which exposed the patient to the risk of a paradoxical embolization, subsequently we anticoagulated with curative doses of low-molecular-weight heparin (LMWH). The non-visualisation of the thrombus on the follow-up TTE raised concerns of a paradoxical embolic event despite the absence of any clinical expression prompting us to perform a whole-body contrast-enhanced CT which came back normal.

The incidental discovery of an atrial septal abnormality should've pushed for a more comprehensive assessement [16] through multiple imaging technics such as a cardiography with agitated saline microbubbles test [17] or an electrocardiography-gated CT using the saline-chaser contrast injection technique [18] allowing to identify an interatrial shunt and to differentiate a patent foramen ovale (PFO) from an atrial septal defect (ASD).

In an Italian multi-center observational nationwide survey, Sabatino et al. [19] reported a 9% incidence of ASD in patients with a confirmed or suspected COVID-19 infection. Simultaneously, cases of PFO revealed by COVID-19 were reported [[20], [21], [22]]. It is important to keep in mind the high prevalence of PFO in the general population [23] and the plausibility of an under-diagnosis in patients with COVID-19 [24]. As for paradoxial embolisms in patients with COVID-19, to our knowledge only 3 cases were reported in the litterature [[25], [26], [27]].

In a narrative review, Mondal et al. [28] reported that venous thromboembolism and pulmonary embolism are respectively the most frequent forms of thromboembolic events related to COVID-19. As for intracardiac thrombosis, our case is the fifth reported within our center [29], among a total of 630 COVID-19 patients admitted and managed since the beginning of the pandemic, joining only few cases reported in the litterature [[30], [31], [32], [33], [34], [35], [36], [37]], alternatively Rastogi et al. [38] reported a total of 17 cardiac thrombi among a 1010 COVID-19 patients hospitalized within a one year timeline.

Various parameters were monitored in order to predict the severity of COVID-19 [39,40], impairement of hemostatic markers (especially elevated D‐dimer and FDP) was widely observed in COVID-19 patients [41], and even linked to a higher incidence of thromboembolic events [42] as well as an unfavourable prognosis [43]. In our case, a gradual decline of D-dimers levels (which were monitored daily since admission) was observed.

The International Society on Thrombosis and Hemostasis (ISTH) recommends the use of prophylactic doses of low molecular weight heparin (LMWH) in all patients (including non-critically ill) hospitalized for COVID-19 in the absence of any contraindications [4] which has been implimented in our center since the very beginning of the pandemic. Alongside its antucoagulant properties, Poterucha et al. [44] layed out the various effects of hearin on inflammation pathaways, thus providing evidence of its anti-inflammatory properties.

The benefice of a curative anticoagulation in patients meeting certain criteria such as the sepsis-induced coagulopathy (SIC) score [45] has been proven, thus making the decision to initiate it much easier. Alternatively The American Society of Hematology (ASH) suggests using curative anticoagulation in patients who develop sudden clinical and laboratory findings consistent with pulmonary embolism, patients with physical findings consistent with thrombosis, and patients with respiratory failure, especially when D-dimer and/or fibrinogen levels are very high [46,47].

As for cardiac thrombosis, treatment options include anticoagulation, thrombolysis, and thrombectomy (surgical or percutaneous). Barrios et al. [48] concluded that in patients with right heart thrombosis associated with pulmonary embolism, there is no significant difference between reperfusion therapy and anticoagulant therapy in terms of mortality and bleeding, in fact, a higher risk of recurrence for reperfusion therapy was reported in comparison with anticoagulation. In our case, the patient upon discussing treatment options opted for curative anticoagulation which led to the lysis of the thrombus.

Regarding limitations, as indicated above, a more comprehensive assessement using targeted imaging technics prevented a more precise diagnosis early-on.

Nonetheless, Follow-up led to a retrospective diagnosis of a functional closure of a PFO due to the resolution of the pulmonary embolism and subsequent normalization of right atrial pressure confirmed by cardiography.

## 4 Conclusion

COVID-19-related thromboembolic events are associated with a higher risk of mortality [3], and are attributed to specific coagulopathy distinctive of COVID-19 [9].

Our case's uniqueness resides in the unfortunate association of a right atrial embolism and an interatrial communication in a young patient with no cardiovadcular risk factors or history which underlines the likelihood of a significant underdiagnosis of atrial septal abonamalities especially given the high prevalence of PFO in the general population, and might account for a higher mortality in COVID-19 patients.

It also highlights the importance of contrast-enhanced imaging and cardiac evaluation in COVID-19 patients which may results in diagnosing more thromboembolic events and subsequent adequate management.

This paper has been reported in line with the SCARE 2020 criteria [5].

## Ethical approval

This is a case report, therefore Ethics committee/IRB approval is not required.

## Sources of funding

This work hasn't received any funding.

## Author contributions

SAMIA BERRICHI: Study conception, Data collection; data analysis; writing & editing.

ZAKARIA BOUAYED: Data collection; data analysis; writing & editing.

KARIMA BENBOUCHTA: Cardiac imaging and analysis.

AMINE KOSSIR: Cardiac imaging and analysis.

NABILA ISMAILI: Supervision and review data validation.

NOHA EL OUAFI: Supervision and review data validation.

HOUSSAM BKIYAR: Supervision and review data validation.

HOUSNI BRAHIM: Supervision and review data validation.

## Registration of research studies

1. Name of the registry:

2. Unique Identifying number or registration ID:

3. Hyperlink to your specific registration (must be publicly accessible and will be checked):

## Guarantor

SAMIA BERRICHI.

ZAKARIA BOUAYED.

## Consent

Written informed consent was obtained from the patient for publication of this case report and accompanying images. A copy of the written consent is available for review by the Editorin-Chief of this journal on request.

## Conflicts of interest

We have no conflicts of interest.

## Appendix A Supplementary data

Multimedia component 1 Fig. 6: TTE apical four-chamber view video showing the large thrombus inside the right cavities protruding into the left atrium. Multimedia component 1
